# 1159. A Phase 2 Drug-Vaccine Interaction Study of AV7909 and Ciprofloxacin or Doxycycline on Antibiotic Pharmacokinetics

**DOI:** 10.1093/ofid/ofad500.999

**Published:** 2023-11-27

**Authors:** Gideon Akintunde, Bojan Drobic, Lisa Bedell, Lu Gan, Julia Kim, Marinda Beah, Isaac Ghinai, Santiago Barona Collado

**Affiliations:** Emergent Biosolutions, Gaithersburg, Maryland; Emergent Biosolutions, Gaithersburg, Maryland; Emergent Biosolutions, Gaithersburg, Maryland; Emergent Biosolutions, Gaithersburg, Maryland; Emergent Biosolutions, Gaithersburg, Maryland; Emergent Biosolutions, Gaithersburg, Maryland; Emergent Biosolutions, Gaithersburg, Maryland; Emergent Biosolutions, Gaithersburg, Maryland

## Abstract

**Background:**

AV7909 (Anthrax Vaccine Adsorbed, Adjuvanted) vaccine was developed for post-exposure prophylaxis (PEP) of disease following suspected or confirmed exposure to *Bacillus anthracis* administered in conjunction with recommended antibacterial regimen. A drug-vaccine interaction study (NCT04067011) was conducted to examine whether co-administration of AV7909 with ciprofloxacin (CIP) or doxycycline (DOX) affects antibiotic pharmacokinetics (PK) or AV7909 immunogenicity in healthy adults.

**Methods:**

A Phase 2, randomized, open-label study was conducted to assess the effects of AV7909 (intramuscularly (IM) at 0, 2 weeks) on PK profiles of orally administered CIP and DOX in healthy adults between 18 and 45 years of age. Participants (n=210) were randomized to receive AV7909+CIP, AV7909+DOX or AV7909 alone. Serum concentrations of CIP and DOX were determined using liquid chromatography with tandem mass spectrometry, while AV7909 immunogenicity was evaluated using 50% neutralizing factor (NF_50_) values generated by a toxin neutralizing antibody (TNA) assay. Steady-state maximum concentration, C_max_ and area under the curve from 0 to 12 hours, AUC_0-12hrs_ for CIP and DOX were calculated. Safety was evaluated by collection of solicited reactogenicity and reports of adverse events (AEs).

**Results:**

Primary PK endpoint for CIP was met, wherein 90% confidence intervals (CIs) of mean ratios for steady-state AUC_0-12h_ [90% CI: 0.89, 1.07] and C_max_ [90% CI: 0.87, 1.08] were contained within the equivalence criteria [0.80, 1.25]. The endpoint was marginally missed for DOX, wherein lower bound (LB) of 90% CI for AUC_0-12h_ [90% CI: 0.82, 1.03] was within the equivalence criteria, but LB of 90% CI for C_max_ (0.79) was below the equivalence criteria [0.80, 1.25]. This result for steady-state DOX C_max_ is likely limited given that its efficacy is mainly AUC-driven. The immunogenicity endpoint was met, wherein LB of two-sided 95% CI of the geometric mean ratio of TNA NF_50_ was >0.5 (0.78 for CIP, 0.81 for DOX). The majority of solicited reactogenicities and AEs were Grade 1 or 2 in severity.

**Conclusion:**

Co-administration of CIP or DOX with AV7909 did not alter the relevant PK parameters of antibacterials or immunogenicity of AV7909 and was well tolerated in healthy adults.

**Disclosures:**

**Gideon Akintunde, Director Clinical Development**, Emergent Biosolutions: Employee **Bojan Drobic, Director Clinical Development**, Emergent Biosolutions: Employee **Lisa Bedell, Director Biostatistic**, Emergent Biosolutions: Employee **Lu Gan, Director Biostatistic**, Emergent Biosolutions: Employee **Julia Kim, Scientist Clinical Development**, Emergent Biosolutions: Employee **Marinda Beah, Manager Clinical Trials Development**, Emergent Biosolutions: Employee **Isaac Ghinai, Medical Director**, Emergent Biosolutions: Employee **Santiago Barona Collado, Medical Director**, Emergent Biosolutions: Employee

